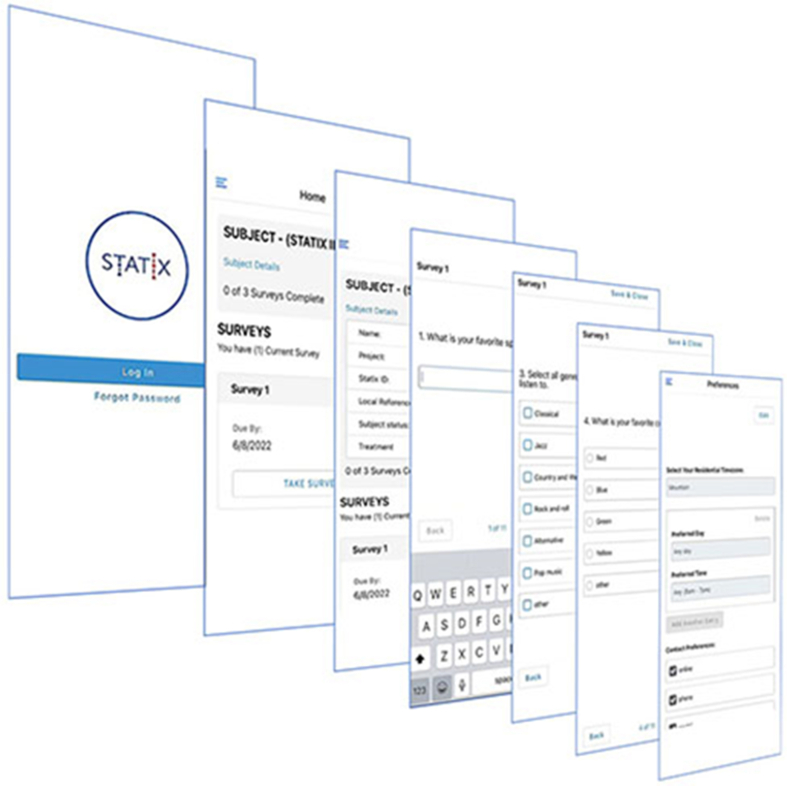# Collecting patient-reported outcomes for the assessment of interventions for pain conditions: Development, accuracy and usability of a customizable mobile app

**DOI:** 10.1016/j.inpm.2024.100427

**Published:** 2024-07-30

**Authors:** Brook I. Martin, Aaron Conger, Taylor Burnham, Daniel Finch, Zachary L. McCormick

**Affiliations:** Department of Orthopaedics, University of Utah, Salt Lake City, UT, USA; STATIX, LLC, Salt Lake City, UT, USA; Department of Physical Medicine and Rehabilitation, University of Utah, Salt Lake City, UT, USA; Department of Orthopaedic Surgery, Tufts University, Boston, MA, USA; STATIX, LLC, Salt Lake City, UT, USA; Department of Physical Medicine and Rehabilitation, University of Utah, Salt Lake City, UT, USA

Dear Editor,

We are writing to describe a novel platform for collection patient reported outcomes (PROs) in the context of interventional pain research. Validated and reliable PROs, such as measures of health-related quality of life or rating scales for pain intensity and disability, can provide valuable information about symptom burden, and health behaviors when assessing pain interventions.^1^ They are directly elicited from patients enrolled in clinical trials, health registries, and quality improvement programs. However, PRO collection can be time consuming, expensive and technically challenging. A 30–40 % rate of non-response, drop-out, and loss to follow-up is common.^2^ Mitigating non-response is best achieved through efficient instrument design, methods to relocate participants when lost, and a multi-modal approach to capturing participant responses via phone, web, mobile surveys, and reply mail surveys.^2^

STATIX, LLC (www.statix.com, Salt Lake City, Utah) recently developed a customizable data platform and mobile app for interventional pain medicine clinical trials to provide PRO/ePRO collection (SUPPLEMENTAL Fig. 1). “Customizable” means an investigator can design survey instruments and specify the timeline for data collection to suit the needs of his or her research. The platform includes a dashboard specifically designed based on a user's role, including site coordinator, administrators, research investigators, data users, survey interviewers, and study participants. Each user is provided only the minimal amount of information needed to perform their function in accordance with the principles of minimum necessity, separation of duties, and least privilege. The platform supports questions of data types that are text, numeric (continuous), mutually exclusive options (e.g. categorical radio buttons), multiple selections (categorical), lists (e.g. medications), dates, and slider bar (continuous) type questions. Additional features include enabling site coordinators to access and monitor study participant progress, automation of reminder letters and emails appropriately timed based on the follow-up schedule defined by the investigator, a pool of phone interviewers who actively contact study participants, and processing postage-paid reply mail surveys to participants. The platform also integrates a mobile app in iOS and Android with “push notifications”, allowing each participant to complete the PRO surveys when they are due via a mobile device (SUPPLEMENTAL Fig. 2). Survey results are saved to the central database in real time. Separate screens within the platform enable password recovery and editing of contact information and contact preferences. The platform is thus a full featured, multi-modal data collection system integrating phone, mailed and web-based, and mobile app surveys.

Data security and privacy were paramount features built into the platform. This includes compliance with the Health Information Portability and Accountability Act (HIPAA), Code of Federal Regulations (CFR) Title 21 Part 11, pertaining to electronic records used in research, and Good Research Practices for ePRO collection, including maintenance of regulatory safeguards in electronic records such as audit trails, user validation, data encryption, backup, and time stamps. These were assessed and documented using the Higher Education Community Vendor Assessment Toolkit (HECVAT), a standardized assessment of cybersecurity and privacy methods for cloud-based service technologies.^3^ Technology standards in HECVAT are adapted from the National Institute of Standards and Technology Cybersecurity Framework (NIST CSF v1.1), Cloud Security Alliance (CSA) Assessments, the Service Organization Control-2 (SOC-2) audits, and the Federal Information Security Modernization Act (FISMA) standards. Completion of this assessment led to us obtaining a vendor status within our institution.

A focus group tested the platform's accuracy, identified errors, and rated its usability. Usability refers to the ease of use, clarity of instruction, design of the user interface, and intuitiveness. It was measured using the System Usability Scale (SUS), a widely used and validated 10-item rating of product ease and acceptance with items such as support, training, and complexity.^4^ The overall accuracy of the data stored was 96 %, with identification of an error in one type of question that was since programmatically resolved. The overall usability score was 94.6 (sd 6.2) and ranging from 82.5 to 100 across the focus group members and corresponding to “excellent” usability.

The demand for PRO/ePRO collection systems is increasing at a 15 % annual growth rate^5^ fueled by the needs of researchers, regulatory agencies’ acceptance of cloud-based and mobile technology in research, and health systems seeking to comply with new reimbursement policies. Because collection of PROs is often prohibitively expensive, technologically challenging, and time consuming, outsourcing PRO/ePRO data collection to dedicated experts utilizing multi-modal collection techniques is likely to be a viable, efficient, and promising solution. Our customizable PRO/ePRO data collection platform responds to this need: it has been successfully and securely implemented in a secure hosting environment and deployed for iOS and Android. The platform is currently being used in four clinical trials, randomizing – in combination - nearly 18,000 patients, and achieving greater than 90 % PRO response.

A scalable platform for customized PRO/ePRO data collection has obvious appeal. It adds convenience for study participants to report outcomes, facilitates more frequent data collection with more contemporaneous assessments, and makes it easier for researchers to use PRO data. This would likely enhance compliance and convenience for participants enrolled in interventional pain medicine trials while improving the response rate and usefulness of data to support ongoing and future research.

Interested readers of Interventional Pain Medicine are invited to contact the authors to learn more about the platform's features and functionality, data architecture, validation study, and usability. This technology may provide a solution for more efficient and cost-effective data collection for studies of interventions for various pain conditions as opposed to traditional, labor-intensive methods.

## Ethics approval and consent to participate

The study was exempted from human subjects’ review by the University of Utah Institutional Review Board, IRB reference number 00164316. All focus group members voluntarily participated in the project.

## Consent for publication

Not applicable.

## Availability of data and materials

Not applicable.

## Generative artificial intelligence or AI-assisted technologies

No generative AI or AI-assisted technologies were used in the content or writing of this manuscript.

## Submission and verification

The authors verify that this work has not been previously published, it not under consideration for publication elsewhere, and that its submission is approved by all authors.

## Informed consent and patient details

Not applicable.

## Funding

This study was partially funded through grants from the University of Utah (1) Department of Physical Medicine and Rehabilitation Tier 4 Pilot Grant Program, and (2) Skaggs Foundation for Research on Spine and Musculoskeletal Seed Grant. These funding bodies had no role in the design of the study, in the collection, analysis, and interpretation of data, or in writing the manuscript.

## Authors’ contributions

BIM oversaw the apps technical development, analyzed the focus group data, and was the major contributor to drafting the manuscript. BIM, AC, TB and ZM contributed to study design and helped with obtaining study funding. All authors read and approved the final manuscript.

## Declaration of competing interest

BIM is the founder and CEO of STATIX, LLC, a paid research consulting firm, discussed herein. ZM receives research funding from Avanos Medical, Boston Scientific, Relievant Medsystems, Saol Therapeutics, Spine Biopharma, SPR Therapeutics, Stratus Medical, NIH, DOD. Consultancies: Avanos Medical, Saol Therapeutics, Stryker, OrthoSon. All other authors declare that they have no competing interests.

## Figures and Tables

**figs1:**
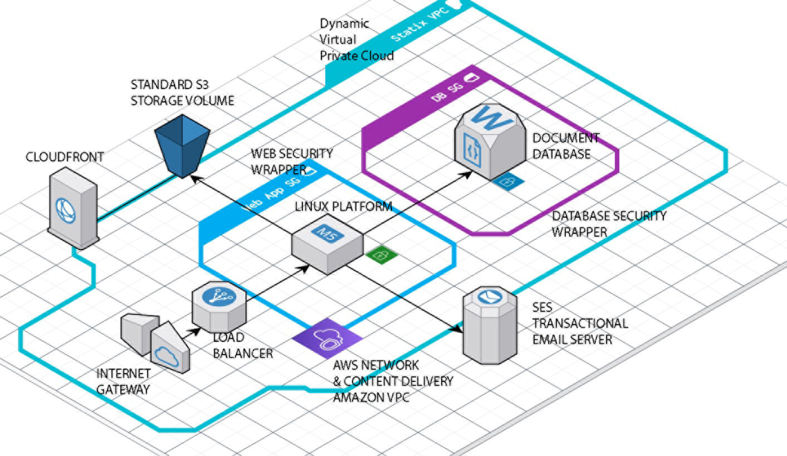


**figs2:**