# Supplementary material for: Collecting patient-reported outcomes for the assessment of interventions for pain conditions: Development, accuracy and usability of a customizable mobile app
Source: Interv Pain Med. 2024 Jul 30;3(3):100427. doi: 10.1016/j.inpm.2024.100427 (PMC11536289; doi:10.1016/j.inpm.2024.100427)
Supplement: Multimedia component 1 [file mmc1.docx]

**APPENDIX A –Studies using STATIX platform.**

The STATIX PRO platform and mobile app are currently being used with three intervention pain medicine clinical trials at the University of Utah. Each trial uses the platform to validate potential subject’s eligibility, to provide a block-stratified, randomized treatment assignment, and to collect patient reported outcomes. Additional details about STATIX can be found at [www.statix.com](http://www.statix.com). The three interventional pain trials are:

- ***A Phase I/II Double-Blinded Randomized Prospective Study of Sterile Amniotic Fluid Filtrate Epidural Injection for the treatment of Lumbosacral Radicular Pain due to Spinal Stenosis (“The SAFE Trial”).*** Sponsored by the University of Utah’s Cell Therapy and Regenerative Medicine Program, this study is a double-blind randomized, prospective trial of an FDA Investigative New Drug (IND) comparing epidural corticosteroid injection (control) to epidural injection of sterile amniotic fluid filtrate in patients with lumbosacral radicular pain due to spinal stenosis (test treatment). STATIX oversees the screening, randomization, and follow-up data collection for the trial, collecting safety and PRO/ePRO data at 12, and 24-hours post injection, as well as 3 and 6-weeks, and 3-, 12-, and 24 months following their first injection.
- ***Conventional or Bipolar Radiofrequency Ablation for the Treatment of Sacroiliac Joint Pain? The COBRA-SIJ study, a Double-blinded, Randomized, Comparative Trial.*** This study is a double-blind, randomized, prospective trial comparing two types of image-guided percutaneous radiofrequency ablation (RFA) for the treatment of sacroiliac joint pain. The study will compare PROs for pain and disability based on randomization to one of two FDA-approved ablation techniques: either bipolar using the Nimbus electrode (N-SIJRFA) or conventional (C-SIJRFA). STATIX collects pain and disability scores as measured by the Oswestry Disability Index, the Patient Global Impression of Pain, and the Numeric Pain Rating Scale at 3, 6, 12, 18, and 24-months following enrollment.
- ***The Effectiveness and Procedural Characteristics of the Trident Multi-tined Cannula for Cervical Medial Branch Radiofrequency Ablation Compared to the Conventional Cannula; A Multi-site, Single Blinded, Randomized Controlled Trial.*** This trial is testing whether a novel Trident cannula used in cervical medial branch radiofrequency ablation (CMBRFA) offers reduced procedural discomfort, procedure time and radiation exposure, without an inferior change in pain and function compared to conventional cannula, Pain and function measures are collected from 120 patients at 2 weeks, and 3, 6, and 24 months.

**APPENDIX B – Technical description of mobile app for ePRO**

The cloud-based platform runs on a robust, secure, scalable architecture hosted by an Amazon Web Service HIPAA-Compliant web architecture (FIGURE 1), which has passed a SOC-2 audit report, an independent examination of security and privacy controls. The key architectural features include: CloudFront, a globally distributed network of proxy servers for content delivery; a Virtual Private Cloud with dynamic Web App security wrappers and an internet gateway supporting EC2 instances on a Linux T3 Platform (Xeon 3.1 Ghz Plantinum 8000 series processor, 2 vCPUs, 8GiB memory, 2,780 Mbps throughput and 10G network performance); Amazon S3 object storage; and an Amazon DocumentDB R5 managed instance inside of a private subnet within the VPC. All required data input fields are validated on both client and server sides. All EC2 instances run the latest patched version of Ubuntu 18.04 LTS. We encrypt data sent through the platform using Secure Sockets Layer (SSL). All data within the AWS environment, including database and log files stored in S3, are encrypted at rest using an AES-256 cipher. We also leveraged managed web application firewall services (WAF), distributed denial-of-service (DDOS) protection tools, and Domain Name Service (DNS) failover and redundancy features provided through CloudFlare in addition to load balancing, auto-scaling, backup, failover, and auto-recovery functionality within the AWS environment. Server configurations and system setup are maintained as code through CloudFormation scripts, and deployments of the code infrastructure have been automated. Whenever possible, multi region deployment of AWS service is utilized to provide geographic diversity and protection against hosting disruption. Following several iterative rounds of design and testing, we successfully deployed the mobile app to the production environment for both iOS and Android platforms. Platform architecture and screenshots of the mobile app are shown below.

The links to the mobile app for study participants are:

Apple App Store: https://apps.apple.com/us/app/statix/id1604450099

Google Play Store: https://play.google.com/store/apps/details?id=com.statix

*Appendix figure 1:* STATIX’s web-enabled content management systems architecture provides scalability and efficiency.


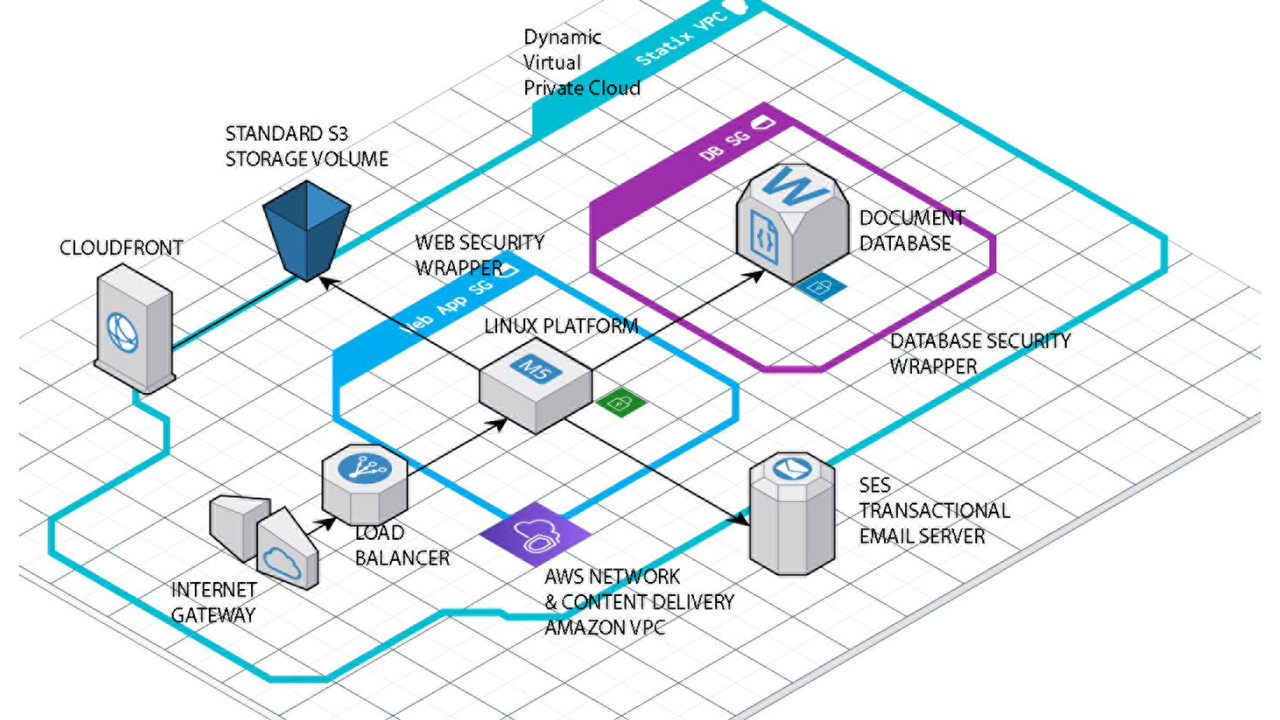


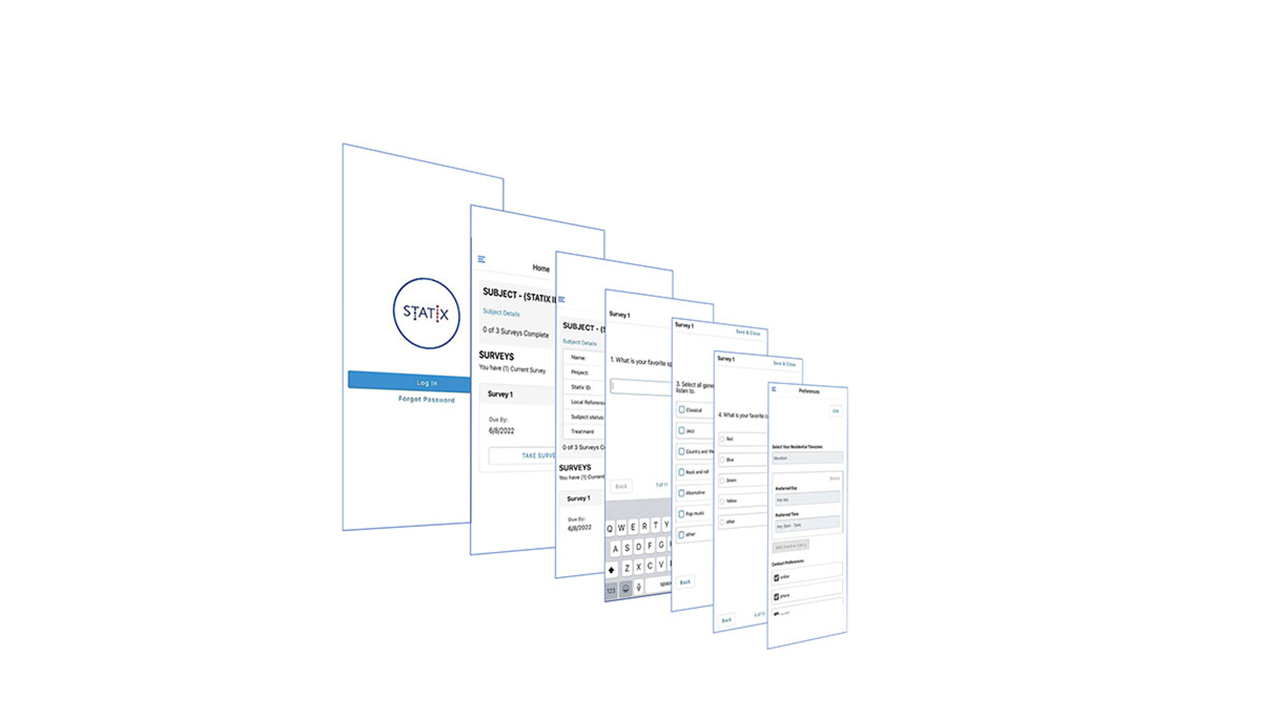


*Appendix figure 2:* Screenshots of STATIX’s ePRO mobile app user interface.

**APPENDIX C – Description of the security of the platform.**

Data security and privacy were paramount features of the app. This includes compliance with the Health Information Portability and Accountability Act (HIPAA), Code of Federal Regulations (CFR) Title 21 Part 11, pertaining to electronic records used in research, and Good Research Practices for ePRO collection, including maintenance of regulatory safeguards in electronic records such as audit trails, user validation, data encryption, backup, and time stamps. We documented the app architecture and security features using the Higher Education Community Vendor Assessment Toolkit (HECVAT), a standardized assessment of cybersecurity and privacy methods for cloud-based service technologies. The HECVAT assessment included over 200 compliance items ranging from adoption of technology to operational and business standards. Technology standards in HECVAT are adapted from the National Institute of Standards and Technology Cybersecurity Framework (NIST CSF v1.1), Cloud Security Alliance (CSA) Assessments, the Service Organization Control-2 (SOC-2) audits, and the Federal Information Security Modernization Act (FISMA) standards. Business standards include establishing HIPAA business associate agreements with contracted agents, confidentiality agreements, interviewer training for the ethical treatment of subjects involved in human research, and documenting business continuity, incident response, physical security, change management, quality assurance, and disaster recovery plans. Upon completion and review of the HECVAT assessment, we successfully obtained approval as a vendor to use our mobile app at the University of Utah.
